# Patient and health-care provider experience of a person-centred, multidisciplinary, psychosocial support and harm reduction programme for patients with harmful use of alcohol and drug-resistant tuberculosis in Minsk, Belarus

**DOI:** 10.1186/s12913-022-08525-x

**Published:** 2022-09-30

**Authors:** Rebecca Elizabeth Harrison, Volha Shyleika, Christian Falkenstein, Ekaterine Garsevanidze, Olga Vishnevskaya, Knut Lonnroth, Öznur Sayakci, Animesh Sinha, Norman Sitali, Alena Skrahina, Beverley Stringer, Cecilio Tan, Htay Thet Mar, Sarah Venis, Dmitri Vetushko, Kerri Viney, Raman Vishneuski, Antonio Isidro Carrion Martin

**Affiliations:** 1 Médecins Sans Frontières, Minsk, Belarus; 2 Department of Global Public Health, Karolinska Institutet, Stockholm, Sweden; 3 Médecins Sans Frontières, London, UK; 4 Médecins Sans Frontières, Berlin, Germany; 5 Republican Scientific and Practical Centre of Pulmonology and Tuberculosis (RSPCPT), Minsk, Republic of Belarus; 6 Médecins Sans Frontières, Moscow, Russia; 7 School of Public Health, The University of Sydney, Sydney, Australia

**Keywords:** MDR/RR-TB (multi-drug or rifampicin resistant tuberculosis), Alcohol use disorder, Harmful use of alcohol, Multi-disciplinary, Person-centred

## Abstract

**Background:**

Tuberculosis (TB) often concentrates in groups of people with complex health and social issues, including alcohol use disorders (AUD). Risk of TB, and poor TB treatment outcomes, are substantially elevated in people who have AUD. Médecins sans Frontières and the Belarus Ministry of Health have worked to improve treatment adherence in patients with multi-drug or rifampicin resistant (MDR/RR)-TB and harmful use of alcohol. In 2016, a person-centred, multidisciplinary, psychosocial support and harm reduction programme delivered by TB doctors, counsellors, psychiatrists, health-educators, and social workers was initiated. In 2020, we described patient and provider experiences within the programme as part of a wider evaluation.

**Methods:**

We recruited 12 patients and 20 health-care workers, using purposive sampling, for in-depth individual interviews and focus group discussions. We used a participant-led, flexible, exploratory approach, enabling participants and the interviewer to shape topics of conversation. Qualitative data were coded manually and analysed thematically. As part of the analysis process, identified themes were shared with health-care worker participants to enable their reflections to be incorporated into the findings.

**Results:**

Key themes related to the patients’ and practitioners experience of having and treating MDRTB with associated complex health and social issues were: fragility and despair and guidance, trust and health. Prejudice and marginalisation were global to both themes. Counsellors and other health workers built a trusting relationship with patients, enabling guidance through a multi-disciplinary approach, which supported patients to achieve their vision of health. This guidance was achieved by a team of social workers, counsellors, doctors and health-educators who provided professional and individualised help for patients’ illnesses, personal or interpersonal problems, administrative tasks, and job searches.

**Conclusions:**

Patients with MDR/RR-TB and harmful use of alcohol faced complex issues during treatment. Our findings describe how person-centred, multi-disciplinary, psychosocial support helped patients in this setting to cope with these challenges and complete the treatment programme. We recommend that these findings are used to: i) inform programmatic changes to further boost the person-centred care nature of this program; and ii) advocate for this type of person-centred care approach to be rolled out across Belarus, and in contexts that face similar challenges.

**Supplementary Information:**

The online version contains supplementary material available at 10.1186/s12913-022-08525-x.

## Background

Despite decreases in global tuberculosis (TB) incidence, multidrug-resistant/rifampicin-resistant TB (MDR/RR-TB) remains challenging, with approximately half a million people infected globally [1]. The Eastern Europe and Central Asia region is particularly affected [1]. The incidence of MDR/RR-TB in Belarus is estimated at 29 per 100,000 population, equating to 4900 incidences per year and also 38% of all new TB incidences are MDR/RR-TB [1]. In many countries, TB is concentrated in groups with complex health and social issues such as homelessness, imprisonment, and alcohol misuse [2]. The risk of TB, and of poor TB treatment outcomes, is substantially elevated in people who drink more than 40 g of alcohol per day, or who have alcohol use disorder (AUD) and who suffer other comorbidities and live in fragile socioeconomic situations [3–6]. In Minsk, Belarus, approximately 40% of patients with MDR/RR-TB have harmful use of alcohol [7].

Evidence for improved packages of care are needed where comorbidities are managed, and health staff and family members need to be sensitised to the complexities of this patient group in order to support these patients to complete TB treatment [8–11]. The effectiveness of person-centred, psychological counselling and educational interventions in improving adherence to TB treatment, including in the case of harmful alcohol use, has been shown, [12–14] and patient-centred care is the 1st pillar in the End TB strategy [15, 16]. However, MDR/RR-TB programmes often tend to focus on preventing TB transmission, case detection, adherence and treatment cure rates, and person-centred approaches to care are usually isolated to pilot projects [17, 18].

In Belarus, the commission of doctors of the treating institution can request the involuntary isolation of TB patients with poor treatment adherence in a hospitalisation centre, [19] which happens particularly frequently in patients with AUD. The number of patients detained has fallen from 341 in 2016 to 188 in 2019, but involuntary isolation remains a concern, and better approaches to support adherence to MDR/RR-TB treatment for patients with AUD are needed [20].

Since 2014, Médecins sans Frontières (MSF) has worked with the Belarus Ministry of Health (MOH) to improve MDR/RR-TB treatment adherence and outcomes and to support a WHO recommendation to reduce involuntary isolation [21] by providing more in-depth out-patient care. In 2016, a person-centred, multi-disciplinary psychosocial support and harm reduction approach (PCMPS programme) was introduced to address the psychosocial needs of people living with drug-resistant tuberculosis who have harmful alcohol use.

We conducted a qualitative study to describe the patient and provider experience of the PCMPS programme for patients with harmful use of alcohol and MDR/RR-TB in Minsk, Belarus.

## Methods

### Study setting

This study took place in Minsk, Belarus, where the estimated number of MDR/RR-TB patients starting treatment every year is around 200. Around 35–45 of these would be enrolled into the PCMPS programme each year. The study took place, within the existing PCMPS programme in the last quarter of 2020.

### Criteria for entry into PCMPS

Newly-diagnosed MDR/RR-TB patients in Minsk were admitted to the in-patient department at the Republic Scientific and Practical Centre of Pulmonology and Phthisiology (RSPCPP) of the National TB Programme. Patients with suspected AUD or harmful alcohol use, according to ICD-10 criteria, [22] were referred to counsellor-educators who assessed the need for psychosocial support. Harmful use of alcohol was considered to be any use of alcohol that is damaging to health [22]. If the counsellor-educator found evidence of AUD or harmful use of alcohol, Pre-screen and AUDIT questionnaires were used. If the patient answered “Yes” to all three pre-screen questions, the Alcohol, Smoking and Substance Involvement Screening Test (ASSIST) tool was then used for multiple substance use assessment. If the patient drinks only alcohol within 1 year, AUDIT C (the first 3 questions of AUDIT) was continued. If AUDIT C was ≥3 in women or ≥ 4 in men, patient was interviewed by asking the remaining questions of AUDIT tool. The classification of scoring for the AUDIT and ASSIST screening are in Table 1. Counsellor-educators would assign a patient’s risk to adherence score (Table 2) based on the outcome of the ASSIST/AUDIT score, and this could be modified based on health and socioeconomic risk factors such as availability of social support, employment, living conditions, previous imprisonment, perception that the patient did not answer the questionnaires honestly, or other mental or physical health co-morbidities.

**Table 1:** AUDIT [23] and ASSIST [24] scoring tool

**Risk**	Low	Moderate	High
**AUDIT Score**	≤ 7	8–15	≥ 16
**ASSIST Score**	Low	Moderate	High
Alcohol	0–10	11–26	≥ 27
Tobacco	0–3	4–26	≥ 27
Drugs	0–3	4–26	≥ 27

**Table 2:** Factors considered in modifications to the risk assessment score

Patient characteristic	Risk assessment
**Socio economic**	
Level of social and family support	Low –moderate - high
Income/ financial stability/ employment situation	Low –moderate - high
History of incarceration	Low –moderate - high
Education level – understanding of the disease and treatment	Low –moderate - high
Housing/ living situation	Low –moderate - high
**Psychiatric or mental health**	
Self-efficacy	Low –moderate – high
Motivation level	Low –moderate – high
Mental health/ psychiatric comorbidities	Yes - No
Forgetfulness, amnesia, dementia	Yes - No
Disruptive sleep	Yes - No
Suicidal ideation/ history	Yes - No
**Health care**	
Distance to DOT provision	Good - Bad
Relationship to health-care providers	Good - Bad
Experience with health-care providers	Good - Bad
General availability of medication	Good - Bad
**Treatment related**	
Comorbidities	Yes - No
Side-effects	Yes - No
Fatigue	Yes - No
Length and complexity of treatment	Yes - No

*DOT* Directly observed treatment

### Summary of PCMPS care package

The care package, provided alongside the MOH’s standard package of MDR/RR-TB care by MOH and MSF staff, includes individual and group counselling, patient education, mental health support, psychiatric care, and social support around 1–3 times per week in person or by phone. TB treatment adherence was ascertained by a nurse through directly observed or video observed treatment. Patients classed as having low risk to adherence received the baseline programme; moderate patients were provided with more intensive counselling; and those at high risk received harm reduction packages, mandatory assessment by a psychiatrist, and anti-craving medication where appropriate. The definitions of care used in the project are in Table 3 and the composition of each care package is in Table 4. Twelve percent of patients in the programme were in involuntary isolation at the time of the study, where they received patient-centred care but could not benefit from all social support elements, such as receiving transport allowance, sanitary kits or support to find a job.

**Table 3:** Care definitions based on the Psychosocial Education and Counselling MSF guidelines 2018 [25]

Activity	Definition
**Patient education:**	Helping the patient to understand their own disease and treatment; enables them to acquire and maintain abilities that allow them to optimally manage their life with this disease.
**Patient counselling**	Aims to help patients find solutions to daily problems that have a negative impact on their adherence to treatment, and to provide emotional support in difficult situations.
**Mental health care**	Involves screening, diagnosing, and treating mental health problems among TB-infected patients.
**Social support**	Encompasses activities aiming to address a weak socio-economic support system.

**Table 4:** Counselling schedule for patients by risk category

Treatment phase	Intervention type	Low risk	Moderate risk	High risk
First 3 months of inpatient treatment (IPD, FHC)	MSF Counselling^a^	1-2x per month	1x per week	1x per week
	MSF Patient contact^b^	1x per month	1x per week	2x per week
	MoH counselling	1x per month	1x per week	2x per week
Rest of inpatient stay (IPD, FHC)	MSF Counselling^a^	1-2x per month	1x per 2 weeks	1x per week
	MSF Patient contact^b^	1x per month	1-2x per 2 weeks	2x per week
	MoH counselliing	1x per month	1x per 2 weeks	2x per week
First 30 days of ambulatory phase (OPD)	MSF Counselling^a^	1-2x per month	1x per week	1x per week
	MSF Patient contact^b^	1x per month	1x per week	2x per week
	MoH counselling	1x per month	1x per week	2x per week
Ambulatory phase to completion of treatment (OPD)	MSF Counselling^a^	1-2x per month	1x per 2 weeks	1x per week
	MSF Patient contact^b^	1x per month	1x per 2 weeks	2x per week
	MoH counselling	1x per month	1x per 2 weeks	2x per week
Discharge phase; last 4 weeks of treatment (all facilities)	MSF Social work counselling	2x per week	1x per week	On request

*IPD* In-patient department, *FHC* Forced hospitalisation centre, *OPD* Out-patient department

^a^Counselling is at least 45 minutes

^b^Patient contact would be a 20 minute or shorter session

### Qualitative methods

We conducted 12 in-depth interviews with patients, and three focus group discussions (FGDs) with 20 health-care providers, in October and November 2020. We used a participant-led, flexible, exploratory approach, enabling participants and the interviewer to shape topics of conversation. We chose the collectivist, multi-vocal focus group as a method as it provides additional insights from group interaction [26]. Patients were asked about their experience of being in the programme, and health-care workers were asked about their experience in programme provision and any implementation issues. The topic guides used can be found in the supplementary material. They were pilot tested on patient counsellors who gave feedback on the suitability for patients. BS trained the interviewer and translators in qualitative methods and the study protocol. All in-depth interviews and FGDs were conducted using English Russian translators, by a female, English speaker, who had previously worked with MSF and who was hired to the programme as an epidemiologist and qualitative researcher. Attention to negative cases was pursued, meaning that contradictory or unexpected findings were actively sought and explored to ensure that predominant themes were a true reflection of participant responses.

### Participant selection

There were around 50 patients in the programme at the time of the study. Participants were purposively selected to represent the full range of perspectives, until data saturation was reached, [27] in the programme based on sex, level of adherence, psychiatric diagnosis, ASSIST and AUDIT scores, and current treatment status: in-patient department (IPD); out-patient department (OPD); involuntary isolation; provider-initiated treatment interruption, or completed treatment. All patients who were considered by the medical team to be too mentally or physically unwell were not invited to interview. Counsellor-educators then offered the opportunity for patients to volunteer to consent to talk to the interviewer and translator, after understanding the aims of the study and content of the interview. All current health-care workers in the programme were invited to take part to ensure a comprehensive range of perspectives.

Due to COVID-19 restrictions, nine interviews and all FGDs were conducted by phone. In-person interviews were conducted in a private space, using infection, prevention and control measures for TB and COVID-19. Two participants had a second interview as it was not possible to finish all the questions in one sitting. Separate FGDs were conducted for MOH and for MSF staff, and a third FGD was held for study investigators who were also implementers. The length of the interviews varied from 15 minutes to 1 hour, and FGDs were between 1 and 2 hours.

### Data preparation and analysis

Audio-recorded interviews and FGDs were transcribed verbatim in Russian then translated into English. Data were sorted and coded manually. Transcripts were analysed thematically, using elements of grounded theory by two co-investigators (RH & BS) using Microsoft Word and Excel to identify patterns in the data; these were constantly compared and refined, with the aim of revealing the experiences of the participants rather than externally imposing interpretations [28]. Data that challenged the general pattern, i.e. negative cases, of the early themes were examined to test these themes and to explain why these situations were different. Themes identified from field notes, taken by the principal investigator, were used to support data analysis. The analyses were discussed and scrutinised until a final coding structure was created. Selected anonymised interview excerpts were drawn out to ensure the individual ‘stories’ were not lost and to explore how themes interrelated [29]. As part of the analysis process, identified themes, but not transcripts, were shared with FGD participants to incorporate their perspectives into the findings. A selection of patients were given a leaflet with summarised results for feedback, some of whom had been interviewed and some not.

## Results

The response rate among patients was 75% (12 interviewed of 16 invited); three of six women (50%) and 9 of 10 men (90%) participated, and age ranged from 32 to 56 years. Reasons for refusal were not given. Not all invited health workers could take part, due to other commitments at work. Providers from one of the clinics did not take part due to having less engagement with the project. Tables 5 and 6 show participant characteristics.

**Table 5:** Characteristics of participants of in-depth interviews (*N* = 12)

	N (%)
**Gender**	
- Female	3 (25.0%)
- Male	9 (75.0%)
**Marital status**	
- In union	4 (33.3%)
- Single	5 (41.7%)
- Widowed/divorced/Separated	3 (25.0%)
**Employment status**	
- Employed	3 (25.0%)
- Unemployed	9 (75.0%)
**History of incarceration**	
- Yes	9 (75.0)
- No	3 (25.0)
**TB classification**	
- Confirmed MDR^a^	3 (25.0)
- Confirmed pre-XDR (FQ)	5 (41.7)
- Confirmed pre-XDR (Inj)	2 (16.7)
- Confirmed XDR^b^	2 (16.7)
**Hepatitis C status**	
- Yes	4 (33.3)
- No	8 (66.7)
**Percent with adherence above 90%**	
- > =90% adherence	9 (75.0)
- < 90% adherence	3 (25.0)
**Previous history of TB**	
- Yes	3 (25.0)
- No	9 (75.0)
**Psychiatric diagnosis**	
- Alcohol dependence	6 (50.0)
- Opioid dependence	1 (8.3)
- Acute alcohol intoxication	2 (16.7)
- Personality disorder	1 (8.3)
- Mild intellectual disabilities	1 (8.3)
**Baseline ASSIST or AUDIT score**	
- Low	2 (18.2)
- Moderate	3 (27.3)
- High	6 (54.5)
**Baseline risk for poor adherence**	
- Moderate risk	5 (41.7)
- High risk	7 (58.3)
**Treatment location at time of interview**	
- Involuntary isolation	2 (18.2)
- In patient	2 (18.2)
- Completed treatment	1 (18.2)
- Out patient	6 (58.3)
- Provider initiated treatment interruption	1 (8.3)

^a^*MDR TB* Multi-drug resistant tuberculosis

^b^*XDR TB* Extensively drug-resistant tuberculosis

**Table 6:** Characteristics of health-care workers who participated in focus group discussions (*N* = 20)

	N (%)
**Gender**	
- Female	13 (65.0)
- Male	7 (35.0)
**Employment**	
- Counsellor-educator	5 (21.7)
- Nurse	4 (17.4)
- Psychiatrist	2 (8.7)
- Psychologist	1 (4.4)
- Social worker	1 (4.4)
- TB doctor	10 (43.5)

**Fig. 1:**
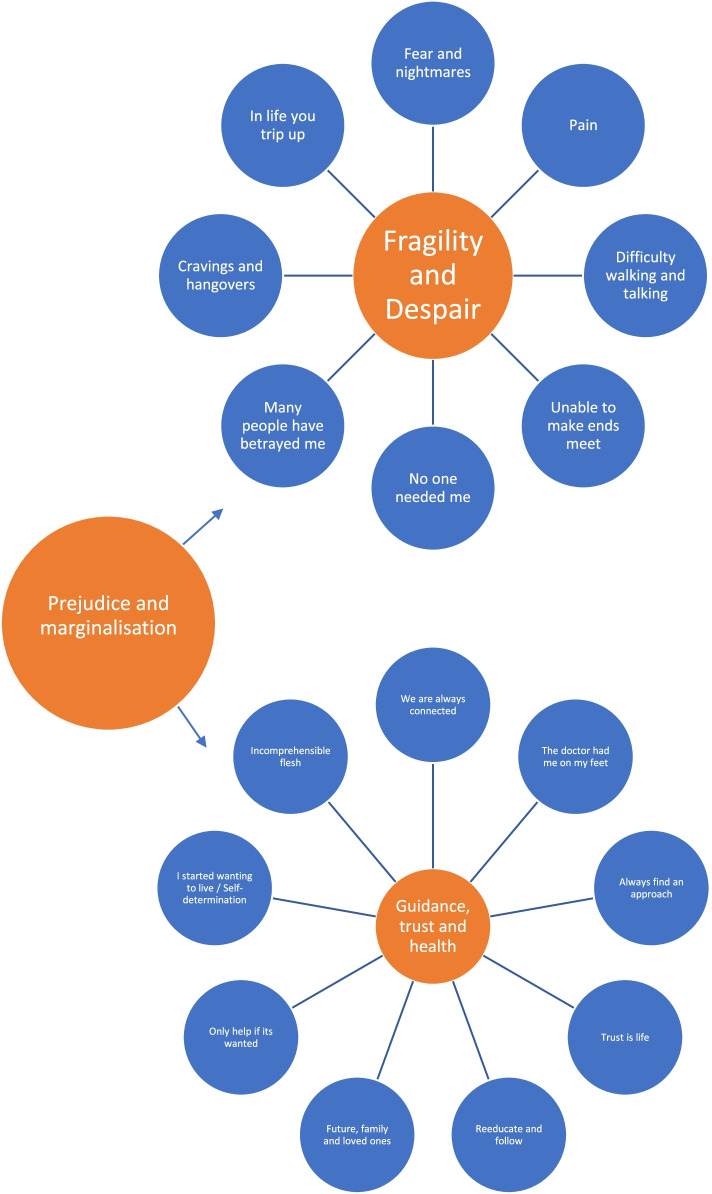
Coding tree

Several key themes emerged, in relation to participant and practitioner experiences of having and treating MDRTB with complex health and social problems: perceptions of fragility and despair, and guidance, trust and health pertinent to the programme. With prejudice and marginalisation described globally across both themes as enabling despair and fragility, with the potential to undermine effective guidance, trust and better health. The coding tree can be found as Fig. 1.

### Experience of fragility and despair

Patients expressed vulnerabilities exacerbated by their physical and mental health and social wellbeing. Anxiety and weakness caused by TB symptoms and side-effects of TB treatment and difficulties in their lives were described, as well as a fear of death and sickness. Although some patients felt healthy, others felt extremely sick from TB. One patient described the feelings of fragility caused by the disease:

“I have a feeling that I want to vomit. Like an old man, you see, it’s just that tuberculosis was like snow on my head”, [Patient F]

Receiving a diagnosis and treatment as an in-patient was described as traumatic by patients and staff. Practitioners described how patients experienced: “rather high anxiety levels at the admission stage because the patients do not understand their diagnosis at all” [MSF Staff]; a patient referred to this stage as “a nightmare” [Patient H]. For many patients, the anxiety experienced at the beginning of treatment was exacerbated by the fear of the effects of the disease on their loved ones. Additionally, some patients were living with HIV infection, hepatitis C, heart disease, or diabetes. These difficulties were recognised by staff:

“If you take tuberculosis, HIV, hepatitis C and social issues all intertwined, it is a heavy load altogether. The chance they will get to the end of such a serious treatment is pretty low” [MSF staff]

Patients in the IPD were often struggling with muscle weakness; others described coughing up sputum and blood. Most experienced treatment side-effects. One patient said: “I feel tired, weak, and I don’t want anything. It’s hard to move. Everything gets hard [after taking the pills]” [Patient J].

The complexity of circumstances for many patients was referred to through their experience of abusing alcohol or other drugs alongside living with TB:

“When I was taking drugs, every day was like Ground-hog Day. Each day was the same. Wake up, look for money, steal something.” [Patient C]

Patients and health-care workers reported that alcohol would be used as a means of dealing with stress, for example: “I washed it down with a drink. I didn’t want to believe I had TB” [Patient E]. The topic of substance abuse wasn’t easy for some participants to discuss or admit to during interviews. For example, one participant said of alcohol abuse: “I know such people, but I’ve got no such challenges. My family comes first for me.” [Patient H]. Counsellor reflections of why participants were not open with alcohol use refer to fear of consequences of being labelled alcohol dependent, which included, for example, potential loss of custody of children.

Economic worries including joblessness or homelessness were an issue for most patients. Some had jobs but they tended to be casual, low-paid manual labour that left them with economic insecurity. Their TB diagnosis prevented some patients having access to employment, but not all were eligible for disability benefits: “I can’t get a regular job. I can’t get a pass from the medical commission to get a job if it can harm me in any way … I don’t understand why they don’t give me a disability group [Patient J]”.

Patients described feelings of physical and emotional isolation, especially where hospitalisation was implemented through the MOH involuntary isolation and treatment system. One patient who had been in and out of prison several times described involuntary isolation and treatment as “a terrible mess. They don’t take patients for humans …. it’s very scary out there. Worse than in prison.” [Patient G].

Many patients described living a lonely and isolated life, with deaths of family members, perceived betrayal, prejudice and marginalisation by family or friends, sometimes due to health issues and diagnoses. One described how his only remaining friend was his dog, and how he felt terribly disappointed by humanity. Another described how this feeling of rejection, prejudice and marginalisation by those around had contributed to his lack of determination to complete treatment or take care of himself: “I faced the situation when the closest people let me down … I was in such a mental state that I felt that no one needed me and they did not care about me.” [Patient A].

### Guidance, trust, and health

The second set of themes relates to the patient and practitioner’s description of guidance, trust and health and its connection to the PCMPS approach. Patients and practitioners stated the importance of being able to build a trusting relationship, and this being a cornerstone for the success of the programme. For those in isolation, counsellors represented relationships that they otherwise might not have:

“Well, it’s always nice seeing them. They always bring something new, new issues, new stories, they tell about what is going on outside the fence, what’s the news. They support and sympathise with us. They are people like us. We also share our news, though little is happening here”, [Patient E]

Counsellor-educators also said that the programme worked through their ability to build trust with a patient. This was often not as simple as following guidelines. Counsellor-educators said that they had to use “… charisma and emotions. You smile or tackle a patient’s personal issues if he’s eager to talk about it. So, you build up trust.” [MSF Staff].

Patients with problems with employment or other social issues were referred to a social worker. If they had other medical problems, the counsellor could help them articulate these and receive the care they needed. This was described as an interconnected experience in which trust was central: “The patient can get help from all sides, and he has people he trusts. He realizes he is not alone in certain processes.” [MSF Staff].

Patients described counsellor-educators as inspirational, trustworthy, and professional. Patients described how counselling helped them deal with conflict with partners, financial or administrative stressors, or fears surrounding TB:

“Here, when I had some issues with my wife, I also contacted [the counsellor-educator]. Well, it is he who helped me. I am glad that I have someone to contact, to talk, to seek advice. Sometimes even not about the disease, but about real life, as they say, situations. So, I’ve got his phone number, we are always connected. I am glad that there is such an organization and I have someone to address.” [Patient A]

MOH doctors felt that counselling as part of the programme meant that patients ‘felt treated as human-beings – which doesn’t normally happen in their lives’ [MOH staff]. Furthermore, one MOH practitioner described how MSF counselling staff could go further and “dig deep into their souls … trying to uncover their deepest fears. And in fact, they often manage to do it.” [MOH staff].

Health-care workers described teamwork and integration of health workers’ differing specialties as essential components of the programme, enabled through having a clear-cut system, finding common ground, and being able to manage crises. Staff met weekly in cross-discipline meetings to discuss patient care:

“That’s where we work together with MOH over the patient’s problem. That’s when we meet together, when we know what’s going on and when we have a single objective to quickly solve his issue. This is quite effective”, [MSF Staff]

In follow up discussions, staff agreed that the programme could potentially go further and integrate more with hepatitis or HIV care, to ensure doctors could treat more than just TB at a single visit, and that patients felt like a human being rather than ‘incomprehensible flesh’ [Patient E]. MOH staff felt that ‘group meetings with different specialists’ [MOH staff] is particularly helpful in patients who are not following guidance on health education.

Most patients described real strength in themselves and a resolve to get through, that was essential for treatment success: “I’ve made up my mind. I’ve got a will power. I want to be cured. I want it to be all right, to go on living”. [Patient E] For some patients, this resilience and determination were learned through surviving army life or previous imprisonment. Determination for many was gained through finding love, or improved relationships with those around them. For example, one patient said “Maybe I’d keep drinking if I’d not met my girlfriend who provided some support …. I have others. I have my mother. I have a wonderful mother. She always supports and helps me” [Patient C].

Many patients talked about the importance of trust: the trust they had in their loved ones, and, in some instances, in practitioners who treated them humanely and with care. One patient said: “I am a distrustful man. Trust nobody. Well. But I trusted him [the doctor]. And I tested him. And when you trust a person, you know … Well, he didn’t give me away to anybody … He treated me like a person whom he is close to. More, he treated me fondly” [Patient K].

### Prejudice and marginalisation

The prejudice, rejection and stigma experienced by patients and their friends and families exacerbated feelings of marginalisation. These feelings were, in some instances, amplified by practitioners who were not following a person centred approach, particularly those treating patients in involuntary isolation. For example, one patient, after 1.5 years of involuntary isolation, said: “It’s not clear at all whether this is a treatment, or I’m in a madhouse, it’s not clear at all” [Patient F]. Some patients described the negative experience of approaches where the doctors only care about adherence to TB treatment, and were not interested in other aspects of a patients’ health:

“But they already have such an attitude that you are not a human being, but a piece of some incomprehensible flesh... [the doctor] says to me ‘The main thing for me is your lungs, and about the rest, I don’t care.” [Patient E]

Some MOH practitioners perceived patients who had a substance abuse and imprisonment history as problematic, stating that: “not just healthcare practitioners are challenged by those patients. They are a burden to other healthcare facilities … as well as to their families and, in fact, to the whole world.” [MOH staff]; while another said “they all want to have fun, they don’t want to work”. [MOH staff].

Not all patients were able to engage with the programme or build trust with the care givers. One MOH staff felt that at least one patient had stopped their TB treatment due to the person-centred approach, as they felt the patients, especially those who had drug problems or a criminal history in some instances were being “indulged” [MOH staff]. Programme psychiatric staff said that patients with other drug use or untreated personality disorders could be the hardest to engage. Patient participants said that there was nothing to be done for patients who were not engaging with the programme until they developed self-determination to get better: “I would say if someone does not need that support, drinks throughout the treatment. You’ll not help them until they start wanting it.” [Patient C].

## Discussion

### Person-centred care to support a group of patients with complex comorbidities

Our study describes the personal situations of a group of patients who often experience poor treatment outcomes, and their perceptions of a programme that is able to engage with them. We illustrate that support and good guidance from a trusted practitioner team, and from reliable loved ones, helps patients to navigate MDR/RR-TB treatment and other challenges faced in everyday life. This was achieved by building trust with patients, leading to better understanding of their complex needs and enabling the provision of holistic care to meet these needs. The importance of “disrupting the cycle of mistrust” between patients and providers has been described in a study of TB care in South Africa [30]. Quality counselling, and care from a multi-disciplinary group of health professionals was largely experienced as positive by study participants. Counsellor-educators built relationships with patients that allowed them to open-up about the entirety of their needs, and helped them meet these through other health providers, with the state or administrative bodies, or with relationships with family or friends. This approach has been shown elsewhere to be beneficial by reducing fragmentation of care, and ensuring communication and coordination between care givers [31]. Other studies have shown that the provision of psychosocial or material support can improve LTFU rates for MDR/RR-TB [32].

### Involuntary isolation and treatment

The programme was able to help address the complex social issues and feelings of marginalisation experienced by patients. However, loneliness and emotional isolation were sometimes worsened by public-health measures involving extended periods of isolation in hospital. Many patients described the psychological difficulties of being locked up for long periods. Involuntary isolation and treatment can lead to psychosocial problems such as loneliness caused by not being able to see a long-term partner, or issues linked to not being able to go about normal social and economic activities [33]. In our study, economic problems were worsened by involuntary isolation, as patients could not keep their employment, or pay for their apartments while in hospital. The impact of lengthy MDR/RR-TB treatment can be worse on those with limited social and financial support networks. In-patient treatment characterised by isolation and focusing mainly on provision of drugs, has been described as worsening feelings of depression and despair, especially in patients with co- and multi-morbidities [34]. Our findings support the further reduction of involuntary isolation and concurrent increase in person-centred support, compatible with WHO recommendation of ambulatory care where possible [35, 36]. Person-centred ambulatory care may initially appear to be at odds with the public health goal of reducing transmission, but if implemented well it should ultimately increase individuals ability to engage sustainably with TB treatment, and thereby decrease transmission [18].

### Room for improvement

Some patients described the negative experience of being treated as “incomprehensible flesh”, where doctors treated their lungs but ignored their other health problems. For some, this de-humanising treatment was painful reminder of the poor treatment they had received elsewhere in their lives.

The PCMPS programme presents a foundational support to include other specialities; for example, coinfections such as HIV treatment at a single doctor’s appointment, or for patients to receive sessions with a group of relevant specialists. The benefits differentiating the person-centred approach could include adherence success for coinfections such HIV, TB, and hepatitis C, especially for injecting drug users, have been reported elsewhere [37–41], and the same benefits could be realised for people who practice harmful use of alcohol. People have also been shown to suffer substantial, negative physical and mental consequences of MDR/RR-TB post-treatment, indicating this intervention could benefit from being extended to include person-centred care after treatment completion [42]. Post-treatment follow-up care may be particularly helpful for those in involuntary isolation, who could not receive all elements of the care package during treatment.

Not all patients were able to engage with the programme, especially those with use of drugs other than alcohol or undiagnosed personality disorders. The concurrence of personality disorder, substance abuse and harmful alcohol use has been well described [43–45]. The program could be further adapted for patients with suspected or confirmed personality disorders, by introducing systematic screening for personality disorder at entry, and by adding an enhanced care package for those patients.

### Limitations

The study design offered a chance for participants to tell their stories to an outsider who had no responsibility in the programme; however, an association of the researcher and translators to programme implementation could not be ruled out, meaning that some participants may have felt compelled to respond positively in interviews. To mitigate this the researcher reflected on role and responsibility throughout and translators were trained in qualitative research methods, and impact on the process was discussed and reflected upon between members of the research team. There may be some bias in responses in that some patients who were interviewed early on in data collection received in person interviews, whereas all later interviews and focus groups were conducted over the phone due to Covid-19. Interviews over the telephone may have restricted the interaction between interviewer and interviewee and removed the possibility for observation. On the other hand, telephone interviews are participant-centred, using a ‘virtual’ communication method that is increasingly common, [46] the telephone may have put some people at ease, allowing them to be more open with socially undesirable responses, or potentially traumatic or sensitive topics [47]. Providers took part in focus group settings, so may not have been comfortable to speak in front of other staff members. Staff who declined to participate may have had different views to those that did participate, However, diverging views were presented by staff, minimising this aspect. Some of the staff members were study investigators, and interviewed separately. All staff were able to feedback results individually to the study investigators, and some did over email, particularly regarding recommendations that arose from the results. We only solicited feedback on the full results from providers, and shared a leaflet describing the key results to patients for feedback. Finally, a standardized patient- or person-centred tool may have helped us to objectively assess user experience and patient satisfaction in comparison to other programmes [48].

## Conclusions

Patients and providers described how the person-centred, holistic approach of the PCMPS programme could lead to an improvement in the ability to cope with life and to complete treatment. Patients valued support from specialised health-care workers who they were able to trust, who treated them as human beings, and who could give them professional help with their illnesses, interpersonal problems, administrative tasks, or finding a job. Health care-workers in the programme appreciated its multidisciplinary nature, which enabled them to solve patients’ problems holistically, after building an initial trusting relationship. The programme should also be integrated further with other specialities such as HIV care. The programme should be adapted in instances where providers were not able to build trust, notably in people who use drugs other than alcohol or are suspected of personality disorder. We recommend that findings from this study are used to inform programmatic changes to further boost the person-centred care nature of this program. Finally, we recommend that the overall positive findings on the experience of this program by patients and health care-workers, are used to advocate for this type of person-centred care approach to be rolled out across Belarus, and in contexts that face similar challenges.

## Supplementary Information

**Additional file 1.**

## Data Availability

The datasets generated and/or analysed during the current study are not publicly available due to the risk of identifying patients or staff in the transcripts, despite de-identification, but are available from the corresponding author on reasonable request.
