# Supplementary material for: Patient and health-care provider experience of a person-centred, multidisciplinary, psychosocial support and harm reduction programme for patients with harmful use of alcohol and drug-resistant tuberculosis in Minsk, Belarus
Source: BMC Health Serv Res. 2022 Sep 30;22:1217. doi: 10.1186/s12913-022-08525-x (PMC9523183; doi:10.1186/s12913-022-08525-x)
Supplement: Supplementary file 1 — Additional file 1. [file 12913_2022_8525_MOESM1_ESM.docx]

# Supplementary material:

Focus Group Discussion for Practitioner group: TOPIC GUIDE

Introduction (5 mins max)

Thank the participants for agreeing to take part in this research

Introduce yourself

Create a relaxed atmosphere

Tell the group “I (We) would like to talk to you about the topics related to the “the person centred care programme for patients with drug resistant tuberculosis” run by Médecins Sans Frontières and the Ministry of health. We are interested in both your particular experiences working in the programme and outlook related to its implementation. This discussion will contribute to a better understanding of how the activities and processes of the comprehensive psychosocial and harm reduction intervention programme contribute to better outcomes for patients.

The interview will take approximately 45 - 60 minutes with anyone in the group deciding to leave at any time if they no longer want to contribute at any time without any consequences.

Make sure the group participants have been informed about the study and have consented verbally to participate in the research

NOTE: check consent for recording and test it is recording.

Go through etiquette when using online platform (e.g. verbal responses, privacy, respect and non-judgment).

| Introduction | - Study aim  - Why invited to participate  - Consent and respect within  the group |  |
| --- | --- | --- |
| History – please share any ideas you have about how this program came about and why different to other types of MDRTB programme (warmer – grand opening question) | Dif with global fund programme  What is an outreach brigade? |  |
| Description of the programme | *We would like you to talk about the programme*  *Describe the different aspects and*  *experience of these?* | How is patient’s health and wellbeing looked after?  What specific issues do you think are  particular to the patients  is it different for women and men |
| Aspects of MSF and MoH | *How may implementation feasibility impact on policy change toward this model of care* | What does the relationship look like  How are the two organisations working together  Describe training support needed to run the programme  Describe quality controls in place linked to procedures skills development |
| Feasibility | *Here we are looking at practicalities – theory versus practice* | Of the components described what happens in practice ?  Staff ratios;  multidisciplinary element;  how is care model realised  competencies needed  ethics as part of practice  safety and confidences |
| Challenges | *What is and is not working* | How do you manage challenges?  What may help overcome these-  Take examples of what support looks like (consistent follow up by one counsellor , family support networks)  What is it like for patients- Can we do better in terms of consistent follow up of patients by someone they trust? |
| Treatment adherence | *From your perspectives describe issues of adherence and how this approach might influence this (as in what affects whether a patient is able to adhere to treatment?)* | Look at responses from different roles (doctor, counsellor etc)  Counsellor perspectives on shame? |
| Success stories | *What does success look like* | Ask for descriptions of success (and failure) related to cases that attribute this programme model to that success  Looking here for learning from challenges too |
| Support for better outcomes | Description from each practitioner or program manager experience of how to support better outcomes | Is there more we should / could be doing for patients who have problems other than alcohol dependence (especially personality disorders)? |
| General observations  moderator |  |  |

**Topic Guide for In-depth Interviews, Person-centred care (PCC) to improve DR-TB treatment outcomes: Assessing a multidisciplinary psychosocial support (PS) and harm reduction intervention in a cohort of MDR/RR-TB patients with harmful use of alcohol in Minsk, Belarus**

1. Background

The in-depth interview for people living with tuberculosis and participating in this feasibility study looking at acceptance of a person-centred care (PCC) programme to improve DR-TB treatment outcomes is carried out in a setting that minimises the influence of the researcher and encourages and stimulates an interviewee. The interview aims to encourage participants to speak about aspects of TB treatment within the social context of their adherence to treatment where specific support to substance abuse is integral to treatment support. The topic guide acts as an aide-memoire for the interviewee, outlining areas or topics of interest framed from results of the survey responses. Interviewees are free to expand on their discussion of any area, or to skip over one completely. In this way the aim of in-depth interviews is to understand more fully interviewee’s perceptions, beliefs, experiences and processes rather than to arrive at a ‘single truth’ across the cohort.

1. Introduction (5 mins max)

Thank the participant for agreeing to take part in this research

Introductions: explain who you are and share some of your experience

Create a relaxed atmosphere; offer the participant something to drink when this is possible

Tell each participant: “I (We) would like to talk to you about your experiences during TB treatment and to what extent the care and approach to difficulties you face is improving this experience. We would like to hear your stories, what you think is working well with regards to how you feel after treatment and the challenges you face. This interview will contribute to a better understanding of how people living with tuberculosis experience treatment in their social context. The interview will take approximately 45 - 60 minutes and can be stopped by you at any time without any consequences. If you would like to continue there is a form we have to complete to check that you have all the information you need before we start. Would you like to continue?

Make sure the participant has been informed and has consented verbally to participate in the research study

NOTE: Turn on the recorder and test it is recording (avoid placing cell phones close to recorder!) Visual aids alongside the topic areas may be used to stimulate perspectives of their health with regard to interviewees’ experience of TB treatment.

3. *Domains: living with TB and substance use; impact on social life; failures with treatment; social network support; adherence events or acceptance toward treatment approaches.

*Domains are chosen based on quantitative survey question framework but may be adapted post initial survey analysis.

| Introduction | -Study aim -Why invited to participate -Consent |
| --- | --- |
| Domain | Prompts |
| Grand opening question: Tell me a little bit about yourself and how you came to be in this TB Programme. | [picking up on anything to be expanded in interviewee’s answer linked to main domains] |
| General health | Views on current general health how do they see their health and social life: what is their story of living with TB- what are their worries and how do they manage these worries about health- previous experience and expectation of TB treatment, what influences the experience – positive and negative? |
| Treatment journey | Treatment life and timetable – what dominates their sense of success to enduring TB each day, week- how often. How treatment and [specific support toward substance abuse] fits into everyday life (challenges, things that help accept and keep to treatment plan)? |
| Taking care of your health | Health-risk behaviours affecting TB treatment [prompts: (e.g. smoking; regular dependency on alcohol or other)].  Understanding of preventative health measures How is taking care of themselves managed – routine? |
| Social life and network of support | What does support feel like? Family support and home life-relationship to support and acceptance of treatment. Knowledge of carer? |
| Adherence events | Main concerns day to day about treatment regime – what might impact adherence that is positive or negative- [food tolerability of treatment; impact of side effect on treatment success; aspects that help or hinder?] |
| Acceptance of programme | Perceived benefits of treatment including all aspects of the person-centred care approach. Other health-promoting measures followed during TB treatment (prompts: e.g. foods, fads, traditions, food supplements). |

4. Any additional information participant would like to share and add that was not mentioned so far.

Thank you again for participating in this interview, any questions?
